# The use of an *in vitro *microneutralization assay to evaluate the potential of recombinant VP5 protein as an antigen for vaccinating against Grass carp reovirus

**DOI:** 10.1186/1743-422X-8-132

**Published:** 2011-03-22

**Authors:** Yongxing He, Hongxu Xu, Qian Yang, Dan Xu, Liqun Lu

**Affiliations:** 1 Key Laboratory of Aquatic Genetic Resources and Utilization/Ministry of Agriculture, Shanghai Ocean University, 201306 Shanghai, China; 2 Department of Medical Diagnostic, Zhongshan School of Medicine, Sun Yat-sen University, Guangzhou 510080, China

## Abstract

**Background:**

Grass carp reovirus (GCRV) is the causative pathogen of grass carp hemorrhagic disease, one of the major diseases damaging grass carp *Ctenopharyngon idellus *breeding industry in China. Prevention and control of the disease is impeded largely due to the lack of research in economic subunit vaccine development. This study aimed to evaluate the potential of viral outer shell protein VP5 as subunit vaccine.

**Methods:**

The *vp5 *gene was isolated from the viral genome through RT-PCR and genetically engineered to express the recombinant VP5 protein in *E coli*. The viral origin of the recombinant protein was confirmed by Western blot analysis with a monoclonal antibody against viral VP5 protein. Polyclonal antibody against the recombinant VP5 protein was prepared from mice. A microneutralization assay was developed to test its neutralizing ability against GCRV infection in cell culture.

**Results:**

The GST-VP5 fusion protein (rVP5) was produced from *E. Coli *with expected molecular weight of 90 kDa. The protein was purified and employed to prepare anti-VP5 polyclonal antibody from mice. The anti-VP5 antibody was found to neutralize GCRV through *in vitro *microneutralization assay and viral progeny quantification analysis.

**Conclusions:**

The present study showed that the viral VP5 protein was involved in viral infection and bacterially-expressed VP5 could be suitable for developing subunit vaccine for the control of GCRV infection.

## Background

Reoviruses are distributed widely in aquatic environments and have been isolated from a wide range of aquatic organisms. Grass carp reovirus (GCRV) is currently one of the most serious pathogens threatening the grass carp *Ctenopharyngon idellus *production with high mortality in China [1]. The virions consist of a double-layered protein capsid containing 11 dsRNA genomic fragments [2]. GCRV was assigned to the genus *Aquareovirus *of the family *Reoviridae *by the international committee on Taxonomy of Viruses (ICTV) in 1991 [3]. It differed from orthoreovirus in a number of characteristics such as absence of an antigenic relationship and unequal numbers of genome segments [4]. To improve the production of grass carp and reduce the economic losses, effective vaccine against GCRV is urgently desired for the fish cultivation industry. However, functional characterization of the encoding proteins of GCRV has been limited due to the lack of research interest of GCRV in regions outside of China. Besides this, the majority of human orthoreovirus infections involve the gastrointestinal and upper respiratory, which are generally asymptomatic [5]. The majority of adults have neutralizing antibodies and no preventative vaccination is needed for the viral infection. Thus, although extensive studies have been conducted on the replication of mammalian reovirus in host cells, little effort has been made to test the vaccine potential of its structural proteins. Fully attenuated apathogenic avian reovirus vaccine did have been developed by serial passage of virus in chicken eggs and chicken embryo fibroblast cultures [6,7]. Outer capsid Sigma C protein had been implicated for the use of potential subunit vaccine against avian reovirus infection [8]. Until now, the only commercial carp vaccine in Asia is an inactivated grass carp reovirus vaccine [9]. Traditional methods, such as attenuation of wild-type viruses to generate live vaccines and formalin-inactivation to produce killed vaccine, are still being employed to develop effective preventive strategy against GCRV in China [10]; the unpopular application of these vaccines at present indicates that further improvement of vaccines in terms of safety, efficacy, manufacturing cost, and field manipulation is essential for the disease control. New advances in molecular biology and biotechnology of virus could help us understand which viral factors are important for induction of strong immunity and lead to new strategies of vaccine design [11].

Identification and production of protective antigens is probably the most feasible strategy towards low-cost vaccines for low-price grass carp. The core of GCRV is composed of five proteins: VP1, VP2, VP3, VP4 and VP6 [12]. A total of 120 VP3 molecules form the spherical inner capsid shell of the GCRV inner core. The outer capsid of GCRV was composed of 200 trimers of VP5-VP7 heterodimers, which were analogous to the μ1_3_σ3_3 _complexes of well-characterized mammalian reovirus but with significant differences in protein structure and low homology. VP7 only shares a very low sequence identity of 12% with its counterpart σ3 of Mammalian reovirus, while the identity between VP5 and μ1 is 24% [13]. The outer capsid proteins of both mammalian reovirus and GCRV were involved in host recognition and attachment during viral replication although not required for efficient viral replication inside the host cells [14,15]. The function of VP7 is unclear at present, while the overall similar structure between VP5 and μ1 suggests a similar functional role of cell membrane penetration during viral entry into host cells for both proteins [13]. Polyclonal antibodies against μ1 and σ3 were reported able to neutralize Mammalian reovirus *in vitro *[14]; thus, among all the five viral structural proteins, VP5 was selected to test its subunit vaccine potential against GCRV in this report.

The aim of present study was to evaluate the potential of VP5 protein as subunit vaccine against GCRV infection through employing *in vitro *microneutralization assays, which has been used successfully for the measurement of neutralizing antibodies to various viruses in vaccine and epidemiologic studies [16]. Information derived from such assays formed the basis for passive immunization strategies against virus infection [17,18]. The investigation also present information on the basic role of VP5 protein in the initial steps of GCRV infection.

## Results

### Expression, purification and immunoblotting analysis of recombinant VP5 protein (rVP5)

**Figure 1:**
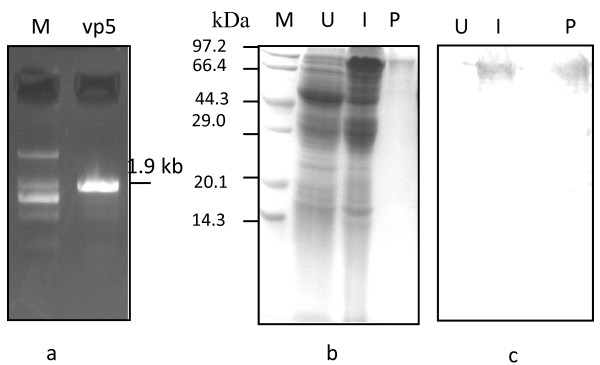
**Cloning, expression, purification and immunoblotting assay of rVP**. **a**, the 1% agarose gel electrophoresis of vp5 gene fragment amplified from GCRV genome by RT-PCR. **b**, 10% SDS-PAGE analysis of induced and purified rVP5. **c**, Immunoblotting assay of rVP5 with monoclonal antibody against VP5 protein. Second antibody was the HRP-conjugated goat-anti-mouse (Sigma). M, Protein marker; U, total cell extract of uninduced bacterial; I, total cell extract of IPTG-induced bacterial; P, purified rVP5.

To produce rVP5 in *E. coli*, the vp5 gene fragment of 1947 bp was isolated through RT-PCR from the purified viral dsRNA genome (Figure 1a). The PCR product was then inserted to the bacterial expression vector pGEX3T-4 for the expression of a GST-VP5 fusion protein, the correct clone bearing the indeed vp5 gene was confirmed by gene sequencing. Expected GST-VP5 fusion protein of about 90 KDa could be induced by IPTG in cultured bacterial (Figure 1b). The expression level of rVP5 was about 10% of the total bacterial protein. The IPTG-induced protein of 50 ng/μl was isolated from the gel for the use of purified antigen to develop polyclonal antibodies against VP5 (Figure 1b). The viral origin of the induced and purified rVP5 was confirmed by immunoblotting assay with a specific monoclonal antibody against viral VP5 protein (Figure 1c).

### Specificity of polyclonal antibody against GCRV virions

**Figure 2:**
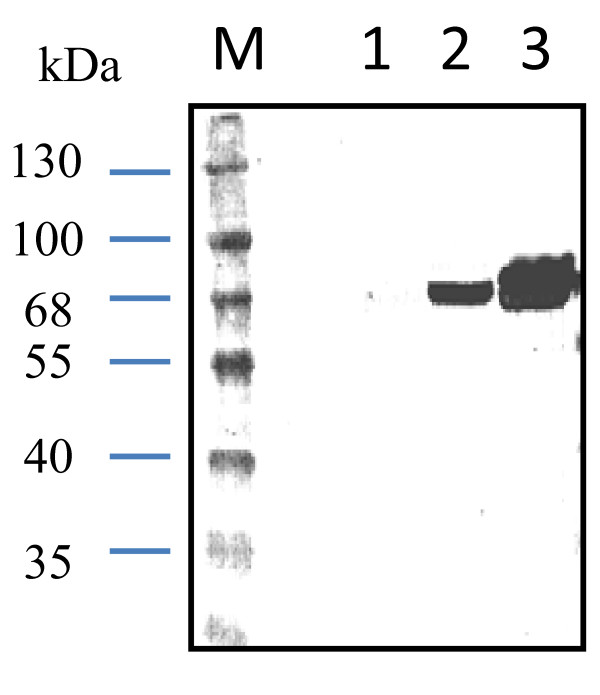
**Immunoblotting assay of GCRV virions with polyclonal antibody anti-VP5**. Mock-infected CIK cell lysate from 10^5 ^cells (lane 1), purified GCRV viral particles of 1 μg (lane 2) and GCRV-infected CIK cell lysate from 10^5 ^cells (lane 3) were subjected for 10% SDS-PAGE analysis and transferred to PVDF membrane. The first antibody used here was the pooled antisera collected from the immunized mice. Second antibody was the HRP-conjugated goat-anti-mouse (Sigma).

To prepare polyclonal antibodies against GCRV VP5 protein, the purified rVP5 was injected into the mice as described in the Materials and Methods. However, it was unclear whether the rVP5 sustained the same antigenicity as viral VP5 protein. Thus, the raised polyclonal antibodies were pooled together, named as anti-VP5 here, for the determination of its specificity toward GCRV viral particles by immunoblotting assay. Figure 2 showed that the pooled polyclonal antibody recognized the viral VP5 protein as revealed by the positive signal at the expected size of about 67 KDa. In contrast, the control serum contained no antibody to recognize viral VP5 protein in the immunoblotting assay (Figure not shown).

### Microneutralization assay and virus titration

**Figure 3:**
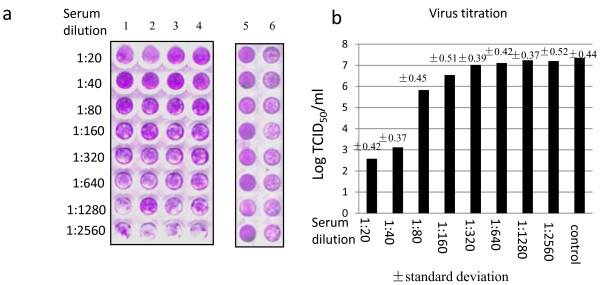
**Neutralization of GCRV by anti-VP5 polyclonal antibody**. **a**, Microneutralization assay. The anti-VP5 polyclonal antibody was tested for four repeats here (Lane 1-4). 50 TCID_50 _virus was used for the assay in Lanes 1-4 and Lane 6. Dilution of anti-VP5 serum was as indicated in the figure (Lanes 1-4), with the diluted preimmunized sera served as control sera here (Lane 6). Mock infected cells with diluted control sera served as positive control (Lane 5). **b**, Virus titration assay. The supernantant of each tested well was subjected for TCID_50 _assay. The data represents the mean value of the four repeats of tested well for each dilution of anti-VP5; the data of Serum control represents the mean value of the 8 test wells served as positive control, in which the control serum was mixed with 50 TCID_50 _virus in the microneutralization assay. Standard deviation of each representative data was as indicated in the chart.

The above results indicated that anti-VP5 polyclonal antibody was able to recognize the viral VP5 protein, but it remained to know whether it contained antibodies that could block the virus infection for evaluation of the potential of rVP5 as subunit vaccine. For this purpose, a microneutralization assay was employed to check the neutralizing ability of the anti-VP5 polyclonal antibody. For more accuracy, the serum dilution was tested in four replicate wells per dilution (Figure 3a). Based on the visual assay result, the neutralization titer of the tested antibody was 1:40.

To confirm the neutralization result, the supernatant of infected cells in the 96 well plate was collected for viral titration assay. Low viral progeny of 10 ^2^-10^3 ^TCID50/ml was observed for the wells infected with GCRV mixed with anti-VP5 sera of 1:20 or 1:40 dilution; while the viral production reached 10^7 ^TCID50/ml for the cells infected with GCRV when mixed with anti-VP5 sera of 1:80 to 1:2560 dilution (Figure 3b).

## Discussion

Grass carp *Ctenopharyngon idellus *culture is an important economic industry for China, which has suffered from GCRV-induced hemorrhagic disease for some years [19]. Although whole virus-inactivated vaccine had been applied in some fish farms for the prevention of the disease, more efficient and economic vaccine approaches were urgently desired for the national-wide control of the viral disease. Subunit vaccine presents an ideal promise for this purpose. The present study aimed to identify the subunit vaccine potential of outer shell protein VP5. As the outer shell protein, VP5 might have key effects on the viral infection, such as recognition and attachment to receptors in the host cell surface, as well as penetration into the host cell membrane during the virus assembly [20]. The fact that anti-VP5 polyclonal antibodies efficiently blocked viral infection here supported that VP5 was involved in viral infection, and posed as a good candidate for the development of subunit vaccine.

The availability of a stable grass carp cell line CIK cells and the strong CPE induced by GCRV infection paved the way for the establishment of an *in vitro *neutralization assay. For the visual microneutralization assay, an optimal working dilution of 50 TCID_50 _virus in 50 μl volume per well was determined in a preliminary logarithmic serial dilution tests. It ensured observable CPE within 48 h postinfection under light microscope in contrast to the uninfected cells. The stained signal of cells in the 96 well plate irreversibly correlated well with the virus-induced CPE for the low dilution of sera (1:20-1:80), which validated the method for neutralization assay. However, for the sera of high dilution (1:160 - 1:2560), the difference of stained signal was not significant, which was reasonable due to the fact that un-neutralized virus replicated quickly in CIK cells. We didn't optimize the production procedure of anti-VP5 antibody from mice in this study. The low neutralizing titer of 1:40 might be largely due to the low titer of anti-VP5 polyclonal antibody raised from mice. To further validate the visual neutralization assay, virus end-point titers were calculated using TCID_50 _assay. The TCID_50 _value of the infected supernatant of each tested well correlated very well with the amount of CPE as showed by the reduction of stained signal of the cell monolayer. It was worthy to note that, in the microneutralization assay, the continued presence of antibody through the course of the assay could neutralize virus released from infected cells.

As far is known, this is the first report on designing a microneutralization assay to test neutralization antibodies against GCRV. The assay could test antibody samples with high throughput since the cell culture, viral infection and staining were all performed in a 96-well format. For further *in vivo *animal experiments to test the protective effect of rVP5 protein, the microneutralization assay on a large scale could still be valuable in addressing the protection mechanism on how and when the neutralization antibody was produced in individual immunized animal. This assay could also be employed for mapping neutralizing epitope of VP5 protein for immunological characterization purpose.

In summary, the gene of GCRV outer shell protein VP5 was cloned and efficiently expressed in *E coli*. to get recombinant protein antigen rVP5, an in vitro microneutralization assay was developed to show that polyclonal antibody against rVP5 was able to block viral infection. The results support the idea that a subunit vaccine based on the rVP5 was possible for the control of GCRV infection in Grass carp *Ctenopharyngon idellus*.

## Methods

### Virus culture

Grass carp *Ctenopharyngon idellus *kidney cells (CIK) were maintained in DMEM medium with 10% fetal bovine serum [21](Zuo et al., 1984). Grass carp reovirus 873 strain (prototype strain) from CCTCC (China center for type culture collection) was used in the study. To propagate the virus, monolayers of CIK were infected with GCRV and incubated for 2-3 days at 28°C. Infected supernatants or cells were harvested when greater than 90% CPE (Cytopathic effect) was observed.

### Viral RNA extraction and RT-PCR reaction

Culture supernatant (250 ml) containing GCRV virions were collected and centrifuged at 60,000 × g for 1 h (SW41 rotor, Beckman). Genomic dsRNA was then extracted from the purified GCRV using a Trizol method (Invitrogen). The dsRNA was separated from contaminating ssRNA by precipitating in 2 M lithium chloride (LiCl). The dsRNA was resuspended in DEPC-treated water and stored at -80°C until use. The reverse transcription was performed with the reverse transcription kit (Takara) using the extracted viral genome template and random primer according to the manufacture's protocol. The synthesis of the cDNA was carried out in a reverse transcriptase reaction mixture at 42°C for 60 min and stopped by the addition of 0.5 M EDTA, pH8.0. The cDNA was used directly for PCR amplification of vp5 gene using Master PCR system (Takara). Vp5 gene was amplified through PCR using primer pairs of TCCCCCGGGGGATGGGGAACGTTCAAACCTCCGT and ATAAGAATGCGGCCGCTTATCACTTGCCGGG CCACAA from the viral genome cDNA mix. Amplification was carried out after denaturation at 94°C for 2 min, followed by 30 cycles of denaturation for 15 s at 95°C, annealing for 30 s at 62°C, extension for 2 min at 68°C and a final extension step for 10 min at 72°C. After sequence confirmation, the vp5 gene fragment was digested by Sma I and Not I before inserting into the corresponding restriction sites of pGEX4T-3 (GE Healthcare) to get recombinant plasmid pGEX-vp5.

### Expression of recombinant VP5 protein and Western blot assay

To express VP5 as glutathione (GST) fusion protein by pGEX-vp5 in DH5α, 100 ml of Luria-Bertani medium containing 100 μg ampicillin/ml was inoculated with 1/100 of an overnight culture containing either of the recombinant plasmids and grown to an optical density of 0.6 at 600 nm. Protein expression was induced with 1 mM IPTG (isopropyl-β-D-thiogalactopyranoside) for 3 h. The bacteria were collected at 6000 × g for 10 min, and the pellet was resuspended in 5 ml of PBS. Protein expression level was judged by SDS-PAGE, through which the ChemiDoc™XRS+ system (Bio-Rad) was employed for the automated quantitative analysis of stained protein samples on PAGE gel. The specificity and viral origin of expressed protein was further analyzed by Western Blot assay using specific monoclonal antiserum again VP5, which was commercially developed using standard methods by injection of specific peptide sequence (291aa-305aa: PRSYRPAFIKPEDAK) of VP5 into mouse at the antibody production facility in AbMart, China. For Western blots, proteins were resolved by SDS-PAGE and transferred to PVDF (Biorad) using standard methods. After blocking in 4% milk and binding of primary antibodies, membranes were washed extensively and incubated with anti-mouse IgG conjugated to alkaline phosphatase. Signal was development by using BCIP and NBT substrates (Sigma).

### Purification of VP5 protein and preparation of polyclonal antibody

The insoluble debris containing the GST-VP5 fusion protein was collected by centrifugation at 5,000 g and washed three times using PBS. Then the total insoluble protein was subjected to SDS-polyacrylamide gel electrophoresis (SDS-PAGE) analysis, then cut from the gel and used for protein purification with PAGE gel protein recovery kit (Sangon Biotech). To generate specific polyclonal antisera towards VP5, groups of 4 adult (6 weeks old) female Balb/c mice were intraperitoneally immunized in a 50% emulsion of Freud's adjuvant with 50 μg of purified recombinant VP5 protein, and boosted twice at 3 weeks interval with the same doses. Immune sera were collected 7 days after the last immunization. Pre-immune serum was collected before immunization and used as a control in neutralization experiments. Immunoglobulin (IgG) antibodies were purified by protein A-sepharose (Promega) and stored at -20°C. Both pre-immune sera and immunized sera were pooled together in the study, which were named as serum control and anti-VP5 serum respectively.

### TCID_50 _(50% tissue culture infective dose) assay

The GCRV titer was determined as TCID_50 _on CIK cells based on a typical cytopathic effect produced by viral infection [22]. CIK cells were maintained as stock cultures in DMEM and re-plated 2 days before infection with GCRV in 96 well plates for TCID_50 _assay. The virus stock was serially diluted with DMEM and then used for infection of CIK. Cell cultures were infected for 1 h, and fed with 200 μl fresh medium. 48 h post infection, 96 well plates were observed under light miscope for typical CPE. The TCID_50 _value was calculated using Reed Muench method.

### Microneutralization assay

The *in vitro *microneutralization assay was performed according to a modified protocol [23]. Cells infected with 50 TCID_50 _virus and cells with mock infection served as positive and negative controls in the assay respectively. The assay medium for serum and virus dilution was the complete growth medium for CIK cells. The sera were inactivated at 56°C for 30 min and serially diluted twofold in a microtiter plate with 50 μl per well. Six serial 2-fold dilutions (1:20 - 1:2560) of serum in a volume of 50 μl were loaded into the plate wells, followed by the addition of 50 TCID_50 _virus. The plates were incubated at 28°C for 1 h. During the incubation period, CIK cells were trypsinized and resuspended at 5 × 10^4 ^cells per ml. Cell suspension (100 μl per well) was added to the plates, the solutions were mixed, and the plates were incubated at 28°C for 48 h. Cells in the 96 well plate were then stained with the dye crystal violet (5% in PBS), which stains only living cells here. If a serum sample contains antibodies that block viral infection, most of the cells will survive and present violet color; if the virus can't be blocked by the serum, cells will be infected, round up and detach from the cell culture plate, thus no violet staining is visible for infected cells. The neutralization titer is expressed as the reciprocal of the highest dilution at which virus infection is blocked.

## Competing interests

The authors declare that they have no competing interests.

## Authors' contributions

LL designed the experiments and drafted the manuscript. YH, HX and QY performed most of the experiments. DX assisted in molecular cloning and protein analysis. All authors have read and approved the final manuscript.
